# Protein expression and genetic variability of canine Can f 1 in golden and Labrador retriever service dogs

**DOI:** 10.1186/s40575-016-0031-3

**Published:** 2016-04-22

**Authors:** Christina Breitenbuecher, Janelle M. Belanger, Kerinne Levy, Paul Mundell, Valerie Fates, Liza Gershony, Thomas R. Famula, Anita M. Oberbauer

**Affiliations:** Department of Animal Science, University of California, One Shields Ave, Davis, CA 95616 USA; Canine Companions for Independence, Santa Rosa, CA USA

**Keywords:** Dog allergen, Can f 1, Service dog, Labrador retriever, Golden retriever

## Abstract

**Background:**

Valued for trainability in diverse tasks, dogs are the primary service animal used to assist individuals with disabilities. Despite their utility, many people in need of service dogs are sensitive to the primary dog allergen, Can f 1, encoded by the *Lipocalin 1* gene (*LCN1*). Several organizations specifically breed service dogs to meet special needs and would like to reduce allergenic potential if possible. In this study, we evaluated the expression of Can f 1 protein and the inherent variability of *LCN1* in two breeds used extensively as service dogs. Saliva samples from equal numbers of male and female Labrador retrievers (*n* = 12), golden retrievers (*n* = 12), and Labrador-golden crosses (*n* = 12) were collected 1 h after the morning meal. Can f 1 protein concentrations in the saliva were measured by ELISA, and the *LCN1* 5′ and 3′ UTRs and exons sequenced.

**Results:**

There was no sex effect (*p* > 0.2) nor time-of-day effect; however, Can f 1 protein levels varied by breed with Labrador retrievers being lower than golden retrievers (3.18 ± 0.51 and 5.35 ± 0.52 μg/ml, respectively, *p* < 0.0075), and the Labrador-golden crosses having intermediate levels (3.77 ± 0.48 μg/ml). Although several novel SNPs were identified in *LCN1*, there were no significant breed-specific sequence differences in the gene and no association of *LCN1* genotypes with Can f 1 expression.

**Conclusions:**

As service dogs, Labrador retrievers likely have lower allergenic potential and, though there were no DNA sequence differences identified, classical genetic selection on the estimated breeding values associated with salivary Can f 1 expression may further reduce that potential.

**Electronic supplementary material:**

The online version of this article (doi:10.1186/s40575-016-0031-3) contains supplementary material, which is available to authorized users.

## Plain english summary

Dogs are valued for their trainability in diverse tasks and have a significant role as service animals used to assist individuals with disabilities. Despite their utility, many people in need of service dogs are sensitive to the primary dog allergen, Can f 1. Organizations that breed service dogs to meet special needs would like to reduce the allergenic potential of their dogs, if possible. In this study, we evaluated the expression of Can f 1 in two dog breeds frequently used for service dogs, the golden retriever and the Labrador retriever. Our goal was to compare salivary Can f 1 expression between the two breeds as well as determine if the Can f 1 gene (*LCN1*) differed at the molecular level and whether a genetic test could be developed to reduce the allergenic potential of service dogs. Males and females had the same levels of the allergen in their saliva however Labrador retrievers had lower Can f 1 levels overall compared to golden retrievers. The genetic sequence for the Can f 1 protein was examined with no breed-specific sequence differences detected. Thus, Labrador retrievers likely have lower allergenic potential than golden retrievers as service dogs because of their lower expression of the primary allergen that evokes human allergies to dogs. Although this difference in expression was not a result of altered DNA in the coding gene, there was inherent variability detected in the expression of the Can f 1 protein among the dogs of a given breed. This difference in expression suggests that classical genetic selection on the estimated breeding values associated with salivary Can f 1 expression may be useful to reduce allergenic potential.

## Background

Allergies to dogs have increased in prevalence presumably as a result of increased dog ownership and sensitization [1–3]. A US Inner City Asthma Study reported that 21 % of asthmatic children also showed allergic reactions when exposed to dog allergens [4]. Dog allergens are present in oral epithelial tissue, hair, dander and urine [5]. Dog allergens produced in oral epithelium can be transferred to the dog’s fur and skin when the dog grooms itself, or into the environment when the dog sheds (reviewed in [6]). These allergens have aerodynamic properties, easily becoming airborne on small dust particles, and can remain suspended in the air for long periods of time [4] or spread throughout the environment even in the absence of the dog [5].

The primary allergen in dog dander and saliva is the protein Can f 1, a member of the lipocalin superfamily that represents the majority of allergens associated with pets (reviewed in [7, 8]). Lipocalins represent the largest mammalian allergen family [9] and comprise the major respiratory allergens from dogs, rats, horses, mice, and cows [10]. Lipocalin family members share several biological properties, including binding and transporting of small hydrophobic molecules such as pheromones, retinol and other steroids, and odorants [9]. As a family, lipocalins consist of 160–230 amino acid residues with a 20 % predicted average amino acid sequence identity [11], sharing three-dimensional structurally conserved regions, despite low amino acid sequence identity [10]. Interestingly, even with conserved structure, each lipocalin protein has unique capacity to induce IgE production [11].

In a previous study done by de Groot et al. [12], 70 % of people allergic to dogs had allergenic reactions to Can f 1, and antibodies against Can f 1 were found in 50–90 % of people who were clinically diagnosed as allergic to dogs [13]. Dander is the predominant cause of mammal-induced allergies in humans [11] because dander contains both sebaceous gland and salivary secretions, including lipocalins.

The expression of Can f 1 in saliva is known to differ across dog breeds. A recent study assessed saliva Can f 1 concentration in single dogs from various dog breeds [14]. In that study, the golden retriever and Dogue de Bordeaux had lower Can f 1 than other breeds. In another study, Can f 1 measured in dander extracts also varied across breeds of dogs: Can f 1 in dander was lower in golden retrievers and Labrador retrievers, and higher in breeds purported to be hypoallergenic [15].

Canine Companions for Independence (CCI), a non-profit organization accredited by Assistance Dogs International, purposely breeds service dogs to assist individuals with disabilities. Since its founding in 1975, nearly 4800 service dogs have been teamed with individuals (http://www.cci.org/site/c.cdKGIRNqEmG/b.4011133/k.787E/Facts_and_FAQs.htm accessed 1/6/2016). CCI has focused, as have many service dog organizations, on the golden retriever and Labrador retriever breeds because of their docile nature, trainability, physical size, and public acceptance (http://www.assistancedogsinternational.org/faq-category/dog-breeds-behaviour/ accessed 1/6/2016). Published research underscores the psychological, social, and physical value of service dogs for people with disabilities [16]. In some cases, disabled individuals may be sensitized to dogs yet wish the benefits of a service dog thereby creating a demand for service dogs with lowered allergenic potential.

The study was done using the golden retrievers, Labrador retrievers, and Labrador-golden crosses bred by CCI to serve as assistance dogs to people with disabilities. To more clearly define expression of the Can f 1 protein in the golden retriever and Labrador retriever, the present study measured its abundance at discrete time points throughout the day. The presence of polymorphisms in *Lipocalin 1* (*LCN1*), the gene that encodes Can f 1, was also evaluated. The goal of this study was to assess if selective breeding approaches could be employed to reduce the allergenic effect of Can f 1 in service dogs. Reducing Can f 1 levels in service dogs could enable quality partnership between the assistance dog and a disabled person who may be sensitive to the Can f 1 allergen. The objective of this study was to quantify Can f 1 protein levels in the CCI dogs, sequence *LCN1* noting any single nucleotide polymorphisms (SNPs), annotated or novel in the CCI breeds and other selected breeds implicated in allergic responses, and then evaluate if there is a correlation between SNPs and protein levels that could allow for the directed selection of dogs with reduced potential to evoke allergic responses.

## Methods

### Animals

All dogs sampled were owned by CCI. To assess whether a single saliva sample would be representative of Can f 1 expression, saliva was collected from five Labrador retrievers, two golden retrievers, and four Labrador-golden crosses at 7:00, 8:30, 12:00, and 16:00. These times were chosen to be prior to the morning meal, an hour after the morning meal, mid-day, and prior to the evening meal. To assess genetic variation in the *LCN1* gene, buccal epithelial cells were collected for DNA extraction from an initial cohort of 4 golden retrievers, 4 Labrador retrievers, and 4 Labrador-golden crosses. An additional 36 dogs (12 golden retrievers, 12 Labrador retrievers, and 12 Labrador-golden crosses with equal numbers of males and females from each breed) were used for saliva and buccal swab epithelial cell collection sampled at 8:30. This time point was selected because it was after the morning meal (one hour) thereby avoiding dietary contamination of the sample and while the dogs were still housed in their kennels prior to daily service training activities at the facility. Overall, 72 % of sampled dogs were neutered and all dogs were over one year of age. Existing DNA samples collected as part of our ongoing studies to identify the genetic basis of canine health disorders were also used to assess genetic variation in the *LCN1* gene for standard poodles (*n* = 12), labradoodles (*n* = 12), and two long-haired, double coated breeds (Belgian shepherds and bearded collies, *n* = 14) selected because of reports that hair can be a major reservoir of Can f 1 and longer-haired dogs may have reduced Can f 1 levels in hair and dander [12, 17]. The genomic sequence of the pug, made available by T-Gen [18], was also compared. All samples were collected in accordance with the approved protocol from the Institutional Animal Care and Use Committee at University of California, Davis and CCI.

### Can f 1 in saliva

To collect saliva samples, a Salimetrics Children’s Swab (Salimetrics State College, PA) was cut in half to create two ~ 2″ long swabs. Swabs were held on the inside bottom of the cheek pocket for one minute, one swab on the left side and one on the right side. A treat was held in front of the dog to encourage salivation. The swabs from each dog were then placed in a 2 ml Swab Storage Tube (Salimetrics, State College, PA) and placed on ice until all saliva samples were collected. Saliva was released from the swabs by centrifugation for 20 min at 3000 rpm and 10 °C. Saliva was pipetted from each sample into a 2 ml low retention tube (Sigma-Aldrich Corp., St. Louis, MO). The entire saliva sample was diluted 1:4 with sterile ddiH_2_O. These were further diluted 1:100 and 1:250 with sterile ddiH_2_O and refrigerated at 4 °C until the next day for analysis.

Total protein in saliva samples was measured using the Bio-Rad protein assay (Bio-Rad, Hercules, CA) according to the manufacturer’s directions. Samples were assayed in triplicate. The *Canis familiaris* allergen, Can f 1, was determined using an ELISA kit (Indoor Biotechnologies, Charlottesville, VA), according to the manufacturer’s directions, and 100 μl of the saliva sample (either diluted 1:100 or 1:250, depending on the concentration of total protein in the saliva sample). These samples were assayed in duplicates.

### DNA collection

Three cytology brushes (Medical Packaging Corporation, Camarillo, CA, USA) were used for each dog for collection of buccal epithelial cells as a source of genomic DNA [19]. Buccal samples were collected an hour after the dog ate or drank to prevent potential particulate contamination from the diet. After swabbing, cytology brushes were placed in their original packaging until DNA was extracted as previously described [20].

### *Lipocalin 1* gene

Primer pairs were designed using Open Primer 3 (http://Frodo.wi.mit.edu/primer3/) based on the Boxer reference genome (http://uswest.ensembl.org/Canis_familiaris/Info/Index) and Canfam3.1 assembly. The six exons and 5′ and 3′ UTR regions of the *LCN1* gene were sequenced as separate amplicons. Amplicons for each sequenced region/exon also contained intronic sequences (Additional file 1: Table S1). The PCR reaction for each sample contained 1× Applied Biosystems taq polymerase buffer II (Applied Biosystems, Carlsbad, CA), 2.5 mM MgCl_2_ (Applied Biosystems), 200 μM dNTPs (Promega, Madison, WI), 1 unit of Amplitaq DNA polymerase (Applied Biosystems) and 0.2 μM of each forward and reverse primer (Fisher Scientific). Correctly sized amplicons were gel purified using the QIAquick Gel Extraction Microcentrifuge Protocol (Qiagen, Redwood City, CA) or ExoSAP-it (USB ExoSAP-IT PCR Product Cleanup, Santa Clara, CA). Purified DNA from the amplicons was sequenced by SimpliSeq DNA sequencing at Quintara Biosciences (South San Francisco, CA).

### Statistical analysis

R software (R Core Team, 2013) was used for the first part of the statistical analyses. Fisher’s Exact Test was used to identify significant breed differences in genotypes among any of the SNPs in the *LCN1* gene. Allelic differences by breed were determined by Chi-squared using Yates continuity correction for datasets with few degrees of freedom. Least squares analysis of variance (PROC GLM, Procedure General Linear Model, SAS version 9.1; SAS Institute Inc., Cary, NC) was used to detect significant differences in the levels of total protein and Can f 1 across the three breed groups, using breed, sex, genotype, and age of dogs as the main effects. Statistical significance was defined as *p* < 0.05 and all data are expressed as mean ± standard error of the mean.

## Results

### Time course

**Fig. 1:**
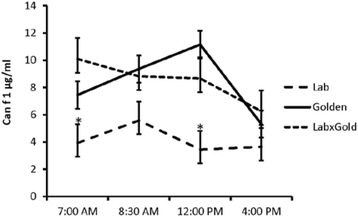
Can f 1 expression over time in three breeds of dogs. Asterisks denote significant differences in mean (± standard error)

Total protein in saliva samples did not significantly differ by time of sampling (2.35 ± 0.22 mg/ml; *p* > 0.08). Similar observations were made for Can f 1 content of saliva collected, which did not significantly differ over the course of 9 h (Table 1). Power calculations demonstrated that the 11 dogs assessed in the present study permitted detection of differences on the order of 3.3 μg/ml, a value consistent with reports of Can f 1 differences [21], indicating a single sample would be sufficient to assess individual Can f 1 levels. When comparing breed contributions, Labrador retrievers had the lowest concentration of Can f 1 both when expressed as total Can f 1 or as Can f 1 corrected for total protein in the sample (Fig. 1 and Table 2, respectively).

**Table 1:** Can f 1 levels in saliva (μg/ml ± standard error) over time (*n* = 11 dogs)

Time of collection	Can f 1
7:00	6.81 ± 1.19
8:30	7.44 ± 1.00
12:00	6.75 ± 1.46
16:00	4.90 ± 0.64

**Table 2:** Can f 1 concentration in saliva (μg/mg ± standard error) over time in Labrador retriever, golden retriever, and Labrador-golden crosses

Time of collection	Labrador (*n* = 4)	Golden (*n* = 2)	Labrador-golden cross (*n* = 4)
7:00	1.97 ± 0.47^a^	3.15 ± 0.75	4.10 ± 0.53
8:30	2.36 ± 0.47	3.48 ± 0.75	3.07 ± 0.53
12:00	1.70 ± 0.47^b^	5.22 ± 0.75	2.83 ± 0.53
16:00	2.04 ± 0.47	2.58 ± 0.75	2.16 ± 0.53

^a^Labrador value significantly different from the Labrador-golden cross

^b^Labrador value significantly different from the golden retriever

### Can f 1

There was no sex difference in salivary Can f 1 expression: 4.32 ± 0.39 and 3.88 ± 0.43 μg/ml for males (*n* = 18) and females (*n* = 18), respectively. There was also no sex difference in total protein measured (1.36 ± 0.10 and 1.08 ± 0.11 mg/ml for males and females, respectively). When comparing the three dog breeds, golden retrievers (*n* = 12), Labrador retrievers (*n* = 12), and Labrador-golden crosses (*n* = 12), Labrador retrievers had significantly lower Can f 1 concentrations (*p* < 0.05) in their saliva than golden retrievers, with Labrador-golden crosses having intermediate levels (Table 3). There were no significant differences in volume of saliva produced by the different breeds.

**Table 3:** Can f 1 protein in saliva and volume of saliva in Labrador retrievers, golden retrievers, and Labrador-golden crosses. Data are presented as mean ± standard error; means carrying different superscripts differ (*p* < 0.05)

Breed	Can f 1 (μg/ml)	Volume of Saliva (ml)
Labrador retriever (*n* = 12)	3.18 ± 0.51^a^	1.23 ± 0.15
Golden retriever (*n* = 12)	5.35 ± 0.52^b^	1.18 ± 0.15
Labrador-golden cross (*n* = 12)	3.77 ± 0.48^ab^	1.40 ± 0.15

### Canine *Lipocalin 1*

In order to test the hypothesis that genetic variability exists, Labrador retrievers (*n* = 4), golden retrievers (*n* = 4), and Labrador-golden crosses (*n* = 4) were initially selected for *LCN1* sequencing. Sequencing confirmed the 36 SNPs recorded in the Ensembl database and identified an additional five novel SNPs detected within the *LCN1* gene, where four of the annotated SNPs exhibited breed-specific differences (*p < 0.05*) in two discrete regions. One region included the 5′ UTR end of the gene and the other was in the 3′ UTR. Thirty-six dogs, for which salivary Can f 1 levels were determined, were then sequenced. Alignment of the sequences showed that *LCN1* is highly conserved across dogs, with none of the SNPs having significantly different allele frequencies for the Labrador and golden retrievers (Table 4). For four SNPs, rs24546658, rs24546659, and rs24546660, rs24565406, Labrador retrievers were fully homozygous, whereas four dogs contributed minor alleles to these SNPs in the golden retrievers (with frequencies of 0.067 for two SNPs and 0.076 for the other two SNPs in golden retrievers). The Labrador–golden crosses were predominantly midpoint in their allele frequencies for all the SNPs, reflecting the combination of the two parental lines. Five novel intronic SNPs were identified in the Labrador and golden retrievers relative to the reference boxer sequence with none having significantly different allele frequencies (Additional file 1: Table S1).

**Table 4:** Variations in Labrador and golden retrievers sequenced for the two *LCN1* regions that had shown initial genomic variation. No variation was determined for SNPs that were fixed for a single allele within the population and have been denoted NA

SNP	CFA location	Alleles (frequency)	Genotypic *P*-values (*n* = 36)	Allele frequency *P*-values (*n* = 36)
rs24546658	9:49713020	C/G (0.933/0.067)	0.0996	0.1071
rs9027939	9:49712904	C/T (0.417/0.583)	0.5272	0.4580
rs24546659	9:49712853	C/G (0.933/0.067)	0.0996	0.1071
rs24546660	9:49712772	A/G (0.067/0.933)	0.0996	0.1071
rs24546661	9:49712734	A/G (0.583/0.417)	0.2689	0.2580
rs24546662	9:49712702	C/T (1/0)	NA	NA
rs8828486	9:49707408	A/C (0.581/0.419)	0.3466	0.2740
rs8828487	9:49707388	A/G (0.565/0.435)	0.3504	0.3412
rs8828488	9:49707260	A/G (0.536/0.464)	0.4022	0.3189
rs24565404	9:49707194	C/T (0.589/0.410)	0.7844	0.7129
rs8828489	9:49707164	C/T (0.482/0.528)	0.5182	0.4514
rs8828490	9:49707115	C/T (0.500/0.500)	0.4127	0.3679
rs24565406	9:49707104	A/G (0.089/0.911)	0.0978	0.0970
rs24565408	9:49707103	A/T (0.054/0.946)	0.2222	0.2217
rs8828491	9:49707082	C/T (0.446/0.554)	0.5066	0.4113

The genotype for each SNP was tested for an association with the expressed level of Can f 1 and no significant relationship was observed. Can f 1 levels in golden retrievers contributing the minor allele to significant SNPs were not statistically different from the mean golden retriever value (4.25 ± 0.14 μg/ml).

Conservation of *LCN1* was assessed in other breeds, including the standard poodle, labradoodle, Belgian shepherd, bearded collie, and pug. In all cases there were few substantive sequence differences identified in the gene or immediate intronic regions. Exons 3, 4, 5, and 6 were conserved in all breeds with the exception of a single bearded collie that had a synonymous SNP in exon 4. Another bearded collie was homozygous for adenosine at CFA 9:49709939 coding for a threonine instead of a proline in exon 1 (c.T21P). The four pugs differed from the remaining breeds in exon 2, at CFA9:49709499, introducing a leucine in place of a serine (c.L52S).

## Discussion

Dog allergens are found in hair, dander, urine, and saliva [5], and may evoke asthmatic and allergic rhinitis responses [13], as well as histamine release [22] in humans. Dander is an imprecise term that includes any tissue sloughed from the body and associated molecules [2] yet dander is often targeted when assessing human exposure to dog allergens because of the deposition of allergens in dander and the accumulation of dander in house dust [2]. The present study focused on salivary Can f 1 because of its abundance in saliva [22] and its contribution to dander. Early studies of dander and salivary sources of dog allergens found both to be potent and equivalent stimulants of human allergic responses [23].

In the Americans with Disabilities Act (ADA) policy related to governmental programs, public areas, and private businesses, the dog is the only recognized service animal (http://www.ada.gov/service_animals_2010.htm [accessed 08.19.15]). With estimates of 8 to nearly 20 % of the human population in the United States self-reporting as being afflicted with dog allergies [24–26] having assistance dogs with reduced capacity to evoke an allergic response would benefit the individual with disabilities who may be allergic to dogs, and others in contact with assistance dogs. Defining Can f 1 protein in the saliva may permit certain breeds or individuals within a breed to be designated as having lower allergenic potential. In this study, the relationship between genetic variability and protein expression of the major allergen found in dog saliva, Can f 1, was investigated to assess the possibility of genetically selecting for reduced expression of Can f 1.

The results from this study corroborate other studies that report equivalent expression of Can f 1 between male and female dogs [15]. The expression profile of Can f 1 did not significantly vary throughout the day nor in relationship to feeding, indicating that a single assessment of salivary protein would be representative of a dog’s propensity to evoke allergies through Can f 1.

The present study did find significant variation in salivary Can f 1 expressed in the two breeds of service dogs used by CCI and their crosses. Another study that used dog saliva to measure Can f 1 levels in golden retrievers, cocker spaniels, and Doberman pinchers also reported great variability of salivary Can f 1 quantities [14], with golden retrievers having the least allergenic protein levels when compared to the other dog breeds assessed. That study, however, evaluated only single individuals from the different breeds and did not include a Labrador retriever.

A study measuring Can f 1 levels in dander extracts from Labrador retrievers, Labradoodles, poodles, Spanish waterdogs, Airedale terriers, and a control group of non-hypoallergenic dog breeds and crossbreds, showed significant differences in variability of Can f 1 both between dog breeds and across individuals within the same breed. Labrador retrievers had the lowest Can f 1 concentration and Poodles had the highest Can f 1 concentration [15] consistent with results of the current study. Another study [21] also using dander extracts likewise demonstrated that golden retrievers had higher Can f 1 concentrations than Labrador retrievers.

Although significant differences in Can f 1 protein expression were detected for golden and Labrador retrievers, there were no significant differences in the underlying gene sequence at either the genotypic or allelic frequency that could account for protein differences. There were also no substantive sequence differences for the additional breeds that were sequenced with the exception of the pug and a single bearded collie. For all but the pug breed, exons 2 through 6 were fully conserved with the reference boxer sequence. The single bearded collie was homozygous at CFA9:49709939 (corresponding to amino acid 21) introducing a polar threonine in place of the hydrophobic proline in the non-conserved region of exon 1, which serves as the signal peptide. Alterations in this region could possibly affect processing of the mature protein, thereby affecting expression. Pugs were heterozygous for a SNP that would substitute a leucine for a serine in exon 2. Numerous β-strands are encoded by exon 2, but the pug mutation, at amino acid 52, was not part of the protein’s conserved tertiary structural elements. Whether this sequence variant would impact Can f 1 expression in the pug was not determined. However, no substantive sequence deviations were associated with differential protein levels in the CCI dogs measured; the same was true for the other breeds reported to have variable Can f 1 expression levels [15, 21]. Thus, no detected underlying sequence variation in *LCN1* accounted for the variability in Can f 1 expression observed in the Labrador and golden retrievers.

An attempt was made to estimate heritability of Can f 1 expression in the studied dogs (data not shown), but the number of related Labrador and golden retrievers having Can f 1 expression values precluded reliable estimation. Given the observed breed differences, a larger number of related dogs with phenotypic expression data may reveal a moderate heritability of Can f 1 that could facilitate classical selection approaches (the need for large sample sizes for effective heritability estimates is reviewed in [27]). Environmental influences could possibly affect the detected Can f 1 concentration, although that seems unlikely given the commonalities of husbandry including diet, housing, and exposures of the dogs at CCI.

Distant non-coding elements of *LCN1* need to be assessed to rule out the role of *LCN1*. Differential *LCN1* expression may be controlled at the level of gene transcription and genetic variability above the level of the *LCN1* gene itself and reflect alterations in the expression, binding, or stability of transcription factors. For example, the 5′ UTR of *LCN1* contains cis-sequences associated with the Pax-4, FoxD3, and CP2 transcription factors [28–30]; the Pax-4 transcription factor has been characterized as promoting development and differentiation of the pancreas [31], FoxD3 is a transcriptional repressor [32] and CP2 has been implicated in the regulation of genes associated with allergy pathways [33]. An additional consideration is the cross reactivity of mammalian lipocalin proteins with IgE assay antibodies [34, 35]. Although the present study used a monoclonal antibody ELISA method specific for Can f 1, there may exist potential cross-reactivity with a different canine lipocalin [36].

In a comprehensive review of pet allergies [2], the literature suggests a threshold response in humans to dog allergens with low exposure being correlated with increased sensitization and elevated exposure being possibly protective. Although selective breeding may reduce allergenic potential in dogs by lowering Can f 1, a greater challenge may be in the human response to the allergen and his/her propensity to mount an allergic response [37, 38]. Another key factor in dog allergies is the human perception of allergy symptoms. Data from a cross-sectional study of children in a metropolitan United States city failed to show an association between levels of dog specific IgE and self-reported allergy symptoms [39] and, in a recent study of dogs described as hypoallergenic, Can f 1 levels were actually higher but the vast majority of owners who self-reported as being allergic to dogs believed their allergy symptoms were reduced with the hypoallergenic dogs [15]. This desire for a hypoallergenic dog has driven the establishment of commercial companies marketing dogs with reduced Can f 1 expression (reviewed in [6]) though the scientific evidence does not support that purported hypoallergenic dogs have lower Can f 1 [15, 40]. Because dog allergens are found on hair [2], dogs that shed less are predicted to have lower allergen contribution to their environment and owners often view hair length as a contributor but the data do not support that supposition [17]. Unfortunately, there is little research that assesses owner perceived allergies with clinician verified pet allergy.

## Conclusions

For agencies partnering assistance dogs with disabled individuals, Labrador retrievers may have a reduced potential to evoke allergies because the breed has significantly lower levels of Can f 1 in saliva and have more compact fur than golden retrievers leading owners to view the dogs as potentially less allergenic. These combined attributes may favor a lower spread of dander and respiratory allergens. Interestingly, the sequence of the *LCN1* gene is highly conserved across individuals within and between breeds. Although no genetic differences were detected for the major dog allergen, individual dogs within a given breed show significant variability in Can f 1 content in saliva and hair [17], suggesting that classical selective breeding approaches using saliva Can f 1 levels as the selection index may be useful in reducing the allergenic potential of service dogs.

## Additional file

Additional file 1: Table S1. Primer sets used for PCR with their corresponding product size and annealing temperature. (DOC 46.5 kb)
