# Supplementary material for: Protein expression and genetic variability of canine Can f 1 in golden and Labrador retriever service dogs
Source: Canine Genet Epidemiol. 2016 Apr 22;3:3. doi: 10.1186/s40575-016-0031-3 (PMC4840867; doi:10.1186/s40575-016-0031-3)
Supplement: Additional file 1: Table S1. — Primer sets used for PCR with their corresponding product size and annealing temperature. (DOC 46.5 kb) [file 40575_2016_31_MOESM1_ESM.doc]

Additional file 1: Table S1. Primer sets used for PCR with their corresponding product size and annealing temperature

| Name | Forward Sequence (5’ – 3’) | Reverse Sequence (5’ – 3’) | Product  Size (bp) | Anneal  Temperature |
| --- | --- | --- | --- | --- |
| 5’ UTR | TCCTGACCCAGGTCAATAGG | AGAAAAGCCAGGGACCTAGC | 568 | 60 |
| Exon 1 | GGAGAGGCCAATTGTCTGTG | GAGCAAGGACACCTGAGGTC | 379 | 60 |
| Exon 2 | CAAGACGGGTCTGTGCCAA | CCACAGAAGATGCCTCACTG | 567 | 62 |
| Exon 3 | GGGTGCAGAGGACAGGAGA | CACCGCACCCCAAGTACTAG | 502 | 60 |
| Exon 4 | CCAAGTACTAGTGGGACGGATCT | ACAGGAACCCAAATGTCAGG | 601 | 60 |
| Exon 5 | ACTACATGCCCACAGCTCCT | GTCTGAAACCTTGGCTGGC | 401 | 62 |
| Exon 6 | CTGCCCAATTGCCTCCAT | CCAATGAGTTGCTTAAAAGATTCA | 405 | 62 |
| 3’UTR | GCAGGTGCAGACATCCAGTA | TTCTGATGGTTCAGGTCCAA | 446 | 60 |
| 3’UTR | CACTTCACATGACCGACTGG | GCTTCTGAAA[TC](http://uswest.ensembl.org/Canis_familiaris/ZMenu/TextSequence?db=core;factorytype=Location;g=ENSCAFG00000019756;r=9:49706594-49713037;t=ENSCAFT00000031415;v=rs8828485;vf=3620105)CCTGGTGA | 564 | 64 |
